# Sharing clinical research data in the United States under the health insurance portability and accountability act and the privacy rule

**DOI:** 10.1186/1745-6215-11-112

**Published:** 2010-11-19

**Authors:** James D Miller

**Affiliations:** 1 Johns Hopkins Bloomberg School of Public Health, Department of Health Policy and Management, Baltimore, Maryland, USA

## Abstract

Sharing of final research data from clinical research is an essential part of the scientific method. The U.S. National Institutes of Health require some grant applications to include plans for sharing final research data, which it defines as the factual materials necessary to document, support, and validate research findings. In the U.S., however, the Privacy Rule adopted under the Health Insurance Portability and Accountability Act impedes the sharing of final research data. In most situations, final research data may be shared only where all information that could possibly be used to identify the subject has been deleted, or where the subject has given authorization for specific research, or an Institutional Review Board has granted a waiver.

## Introduction

For the original investigators in a clinical trial to share their final research data with independent investigators to permit them to build upon or reproduce the original investigators' conclusions is an essential part of the scientific method [1]. The importance of sharing is reflected by evidence that published reports from investigators who share their final research data have significantly more citations compared to reports without sharing [2]. In the U.S., the Privacy Rule [3] adopted under the Health Insurance Portability and Accountability Act of 1996 (HIPAA) [4], impedes the sharing of final research data.

## Discussion

### Background

The sharing of final research data is a long-standing issue, but it came to the forefront in February 2001when *Nature *and *Science *each published draft sequences of the human genome [5,6]. The draft sequence in *Nature*, which was the result of research by the International Human Genome Sequence Consortium, was deposited in GenBank, a publicly available databank, but the sequence in *Science*, which resulted from a commercial enterprise, was deposited only in the commercial enterprise's website, with some restrictions on access. As the U.S. National Research Council noted, the restrictions on access to the sequence reported in *Science *"provoked considerable debate in the life-sciences communities"[7].

Despite this debate, Savage and Vickers, eight years later, found that only one in ten of investigators who published in two journals with "exceedingly" explicit requirements for data sharing actually shared data in response to a request from an independent investigator [8]. This finding is consistent with survey research of life scientists, which showed a perception that sharing of data has become more problematic over the past two decades [7].

The U.S. National Institutes of Health (NIH) have adopted a formal data sharing policy [9], pursuant to which the NIH requires applications for grants of more than $500,000 in annual direct costs to include either a plan for sharing final research data from the research conducted under the grant, or an explanation of why data sharing is "not possible." In particular, NIH "recognizes the need to protect patentable and other proprietary data" in some circumstances. Reviewers of grant applications do not consider the data sharing plan in evaluating the scientific merit and priority of an application. Instead, the NIH program staff addresses the proposed data sharing plan or the explanation of why data sharing is not possible.

In its data sharing policy, the NIH defines "final research data" to mean the "recorded factual materials commonly accepted in the scientific community as necessary to document, support, and validate research findings." Final research data, according to NIH, "does not mean summary statistics or tables; rather it means the data on which summary statistics and tables are based," which usually will be a "computerized dataset." Therefore, final research data will not include case report forms or other "clinical source documents."

NIH apparently does not collect, or at least does not make public, data on what proportion of its grants that are subject to the data sharing policy actually result in the sharing of final research data. Piwowar and Chapman, [10] using a sample of published papers identified by Ochsner et al [11] that reported on DNA microarray research, estimated that, of the papers that appeared to be subject to the NIH data sharing policy (*n *= 61), only 52% referred in the published article to deposition of the microarray dataset in a public depository or elsewhere. While Piwowar and Chapman cautioned that their estimates must be considered preliminary, they found it surprising that the data sharing policy did not apply to a greater proportion of the NIH funded studies in the Ochsner sample. They suggested both expanding the inclusion criteria for triggering the data sharing policy and requiring researchers to cite an open-access database accession number in their published papers and future grant requests. These are valuable suggestions.

### The Privacy Rule

The Privacy Rule is an administrative regulation adopted by the U.S. Department of Health and Human Services (HHS) pursuant to HIPAA. This regulation places stringent and complex limits on the use and disclosure of health information about individuals by many health care providers. Since clinical trials typically take place as part of the provision of health care to individuals, the Privacy Rule has a direct and important effect on the sharing of clinical research data.

The Privacy Rule applies to "Protected Health Information" (PHI), which is "individually identifiable health information" that is "transmitted or maintained" in an electronic media or "any other form or medium." In the Rule, "individually identifiable health information" is defined to mean information created or received by a health care provider (or certain other entities) that:

• relates to an individual's health, or the provision of health care to that individual, including demographic information; and that

• could be used to identify the individual [12].

As an example, the information in the Case Report Forms (CRF) used in many clinical trials might in some circumstances constitute PHI.

A "covered entity" under the Privacy Rule "may not use or disclose protected health information except as permitted or required" by the Privacy Rule [13]. In the Rule, a "covered entity" is defined to include a health care provider "who transmits any health information in electronic form" for certain standard transactions such as claims and benefit eligibility inquiries [14]. (The definition of "covered entity" includes some entities other than health care providers as well.) As a practical matter, most health care providers in the U.S. are "covered entities" and thus subject to the Privacy Rule.

The Privacy Rule contains specific provisions governing the use or disclosure of PHI by covered entities for the purpose of research. A covered entity may conduct research using PHI, or may disclose PHI to an investigator for the purpose of research, in five situations, as follows:

• Where the individual who is the subject of the PHI has authorized in writing the use of the PHI in specific research [15];

• Where the covered entity receives documentation that an Institutional Review Board (IRB) has waived the requirement of an authorization, or has approved the alteration of an existing authorization [16], provided that the IRB meets the detailed standards set forth in the Privacy Rule [17];

• Where the investigator represents to the covered entity that the PHI will be used "solely" to prepare a research protocol or for other purposes "preparatory to research" (such as to aid in study recruitment); that the PHI will not be removed from the covered entity; and that the PHI is "necessary" for these research purposes [18];

• Where the investigator represents to the covered entity that the research is "solely" on the PHI of decedents, and that the PHI is "necessary" for this research [19];

• Finally, a covered entity may disclose for research purposes a "limited data set" of PHI, with certain "direct identifiers" excluded, provided that the covered entity obtains a "data use agreement" with the investigator that the limited data set will be used or disclosed "for limited purposes" [20].

In the context of clinical research, probably the most common means of using and disclosing PHI is by means of an authorization from each subject in the trial. HHS, however, has interpreted the Privacy Rule to require that authorizations for research be limited to specific research that is described in the authorization. This interpretation means that using PHI in future research requires the investigator to obtain a waiver from the IRB, or to obtain a new authorization from each individual subject, either of which is burdensome [21]. The Institute of Medicine, among others, has criticized this interpretation by HHS on the ground that in some circumstances it limits the sharing of clinical data [22,23].

In July 2010, HHS issued a statement that it is considering revising its interpretation and requested comments from interested parties [24]. HHS is considering several alternatives, including that the future research be "adequately described" in the authorization, or that the Privacy Rule be amended to require authorizations to contain specified statements concerning future research. HHS should adopt a revised interpretation that fosters the sharing of data for future research.

### 'De-Identified' Health Information

Since the Privacy Rule protects PHI, no authorization from research subjects is required where PHI has been 'de-identified' as provided in the Privacy Rule so that it is no longer PHI. Covered entities are specifically permitted to use PHI to create de-identified information that is no longer PHI and that accordingly may be freely disclosed and shared [25].

The Privacy Rule permits covered entities to de-identify PHI in two alternative ways [26]. First, a covered entity may have a statistician "with knowledge of methods for rendering information not individually identifiable" determine that the "risk is very small" that the information could be used together with "other reasonably available information" to identify the individual who is the subject of the de-identified information. This method is used sometimes by commercial providers of health informatics data.

Second, a covered entity may remove eighteen specific identifiers listed in the Privacy Rule [27], provided that the covered entity does not have "actual knowledge" that the de-identified information still could be used to identify an individual who is the subject of the information. For purposes of sharing data from a clinical trial, perhaps the most problematic of the eighteen specified identifiers is that "[a]ll elements of dates, including birth dates, admission date, discharge date, [and] date of death" must be removed from the PHI. Hrynaszkiewicz et al have proposed replacing each actual date with a fictitious date derived by adding or subtracting a random number of days from the actual date [28].

### Enforcement of the Privacy Rule

HIPAA and the Privacy Rule do not grant private parties a right to recover money damages from, or an injunction against, a covered entity. [29]. Until 2009, only HHS had authority to enforce HIPAA, including the Privacy Rule, but it tended to do so in administrative proceedings, rather than through the courts. While HHS retains its authority to seek monetary and criminal penalties for HIPAA violations, in 2009 Congress gave the Attorneys General of the States authority to enforce HIPAA and the Privacy Rule by filing civil actions for monetary penalties in federal court [30]. The early experience with this new authority suggests that some State Attorneys General will be aggressive in taking action against covered entities that use or disclose PHI in violation of the Privacy Rule, even when the use or disclosure is plainly inadvertent [31].

The Privacy Rule does not replace (or 'preempt') State laws that are more stringent than the Privacy Rule in protecting individual health information [32,33]. Thus, even an investigator who scrupulously complies with the Privacy Rule could face litigation brought under State law. An important example is the Havasupai Indian Tribe claims against Arizona State University (ASU) [34]. In 2004, the Tribe began a lawsuit against ASU in which the Tribe alleged that researchers at ASU had collected blood samples from members of the Tribe for research into the genetic basis of diabetes but subsequently had used the blood samples in a wide range of research, including migration, inbreeding and schizophrenia that were not directly relevant to diabetes. In addition, the Tribe alleged that the ASU researchers had shared the blood samples with researchers at other institutions. The consent form signed by some members of the Tribe in the early 1990's stated that the blood samples would be used to "study the causes of behavioral/medical disorders," but the Tribe alleged that members of the Tribe were told orally and in writing that the research was on the potential genetic cause of diabetes, which is prevalent among Tribal members [35]. The research apparently was approved by the ASU Institutional Review Board (IRB) in 1991, but the IRB subsequently, and routinely, discarded the records of its approval of the research.

In its lawsuit, the Havasupai Tribe asserted various claims, including fraud and invasion of privacy, under Arizona state law [36].

In 2008, the Arizona Court of Appeals ruled in favor of the Tribe on a technical legal issue, rather than on the merits, but the court's opinion appeared to recognize that the Tribe might have a valid claim on the merits under Arizona law [37]. ASU recently settled the litigation with the Tribe in exchange for an apology and a payment of $700,000 divided among 41 members of the Tribe [34].

## Conclusion

HIPAA and the Privacy Rule impose stringent limitations on the sharing of final research data that constitute PHI. Even where individuals who participate as subjects in clinical research sign written authorizations for the use of their PHI in research, the authorizations are valid only for the specific research described in the authorization, not future research. While PHI may be de-identified so that it is no longer PHI, and therefore may be freely disclosed and shared, the requirement that dates be deleted, and perhaps other required deletions as well, may limit the value of de-identified data in future research.

## Competing interests

The authors declare that they have no competing interests.

## Authors' contributions

The author is the sole author of this Commentary. The author received no compensation or funding for the preparation of this Commentary.

## Author's Information

The author took his law degree from the Yale Law School (1975) and his master's degree in public health from the Johns Hopkins Bloomberg School of Public Health (2008). He engaged in the private practice of law in Washington, D.C. (1979-2006), where he represented pharmaceutical and medical device companies in matters relating to the U.S. Food and Drug Administration. He is a Visiting Scholar in the Department of Health Policy and Management at the Johns Hopkins Bloomberg School of Public Health (2008-present).
